# Global patterns of melanoma, burden attributable to ultraviolet radiation exposure, and projections to 2050 across 185 countries: A population-based study with global modeling

**DOI:** 10.1016/j.jdin.2026.05.026

**Published:** 2026-06-12

**Authors:** Jiyeon Oh, Soeun Kim, Seung Ha Hwang, Yejun Son, Jinseok Lee, Jaewon Kim, Guillermo F. López Sánchez, Damiano Pizzol, Jiyoung Hwang, Dong Keon Yon

**Affiliations:** a Center for Digital Health, Medical Science Research Institute, Kyung Hee University Medical Center, Kyung Hee University College of Medicine, Seoul, South Korea; b Department of Medicine, Kyung Hee University College of Medicine, Seoul, South Korea; c Department of Precision Medicine, Kyung Hee University College of Medicine, Seoul, South Korea; d Software Societal Systems Department, School of Computer Science, Carnegie Mellon University, Pittsburgh, Pennsylvania; e Department of Biomedical Engineering, Kyung Hee University, Yongin, South Korea; f Department of Electronics and Information Convergence Engineering, Kyung Hee University, Yongin, South Korea; g Division of Preventive Medicine and Public Health, Department of Public Health Sciences, School of Medicine, University of Murcia, Murcia, Spain; h Health Unit, Eni, San Donato Milanese, Italy; i Health Unit, Eni, Maputo, Mozambique; j Department of Pediatrics, Kyung Hee University Medical Center, Kyung Hee University College of Medicine, Seoul, South Korea

**Keywords:** GLOBOCAN, melanoma, projections, ultraviolet radiation

## Abstract

**Background:**

Ultraviolet radiation (UVR) is a well-established modifiable risk factor for malignant melanoma, yet global estimates of its attributable burden remain limited.

**Objectives:**

To provide up-to-date global estimates of the melanoma burden and quantify the contribution of UVR.

**Methods:**

We examined melanoma burden in 2022, the most recent ten-year trends, and projected estimates for 2050 by sex, age, and Human Development Index (HDI) using GLOBOCAN data, CI5plus, and the World Health Organization Mortality Databases. As a secondary analysis, population attributable fractions for UVR exposure were calculated for 2022 and 2050, using sex-specific reference populations with low exposure, incorporating demographic changes in the projections.

**Results:**

In 2022, an estimated 330,000 new melanoma cases were reported, with ∼88% attributable to UVR using the fifth percentile reference. Incidence, overall and UVR-attributable, was highest in high-HDI countries, especially Oceania and Northern Europe, while mortality-to-incidence ratios were highest in low-HDI settings. By 2050, UVR-attributable cases are projected to rise, with the steepest growth in low- and medium-HDI settings.

**Limitations:**

Models are subject to uncertainties in cancer registration, especially in low-HDI regions.

**Conclusion:**

Melanoma remains largely UVR-attributable, with substantial regional disparities. The projected increase in UVR-attributable melanoma burden in lower-HDI settings underscores the urgent need for region-specific prevention strategies.

**Capsule Summary**

• This study investigated the current and future burden of melanoma, underscoring their disparities across Human Development Index levels.
• This study highlighted the importance of targeted prevention and the potential to reduce the global melanoma burden through effective UVR exposure mitigation.

## Introduction

Malignant melanoma poses an increasing public health challenge, particularly in high-income countries with predominantly fair-skinned populations.^1^^,^^2^ While not the most common skin cancer, melanoma has a higher mortality-to-incidence (M:I) ratio than non-melanoma skin cancer^3^ due to its aggressive nature and metastatic potential.^4^ In 2022, it ranked among the top 20 most incident cancers globally.^3^ Its burden remains highly heterogeneous, reflecting disparities in skin phenotype, ultraviolet radiation (UVR) exposure, preventive measures, early detection, and healthcare.^5^

Globally, the melanoma burden is primarily driven by modifiable environmental and behavioral exposures, particularly UVR, both solar and artificial. Patterns of UVR exposure vary across populations, shaped by sun-related behaviors, cultural norms, and ambient radiation, making it challenging to estimate UVR-attributable burden. While recent studies have quantified the current global burden of melanoma attributable to UVR using GLOBOCAN 2022 data,^6^ no prior research has projected the future UVR-attributable burden. To our knowledge, our study is the first to provide such forecasts, offering novel insights into the potential trajectory of UVR-related melanoma worldwide.^7^^,^^8^

To address these gaps, this study presents a comprehensive assessment of melanoma burden at the global, regional, and national levels. Specifically, we provide (1) updated estimates of melanoma burden from GLOBOCAN 2022, part of the Global Cancer Observatory maintained by the International Agency for Research on Cancer; (2) current and future estimates of the burden attributable to UVR; (3) most recent 10-year trends in incidence and mortality up to 2023; and (4) projections through 2050 under demographic-based scenarios. The findings by geographic region, Human Development Index (HDI), and age group will support evidence-based decision-making and policy development.

## Methods

### Data sources

Estimates of melanoma of the skin (International Classification of Diseases 10th revision code, C43) were retrieved from GLOBOCAN 2022, part of the Global Cancer Observatory maintained by the International Agency for Research on Cancer.^3^ GLOBOCAN provides modeled estimates of cancer incidence and mortality for 185 countries/territories, stratified by sex and age, using the best available national data combined with statistical modeling where registry coverage is limited, particularly in low- and middle-HDI settings.^7^ Because melanoma risk varies strongly by skin pigmentation, our country-level comparisons may partly reflect underlying phenotype distributions, which are not available for adjustment in GLOBOCAN. Country-specific population estimates for 2022 were extracted from the United Nations World Population Prospects.

To examine temporal trends, annual incidence data were obtained from the Cancer Incidence in 5 Continents Plus (CI5plus) database, which comprises 108 population-based cancer registries with relatively high-quality standards, and annual mortality data were retrieved from the World Health Organization (WHO) Mortality Database, which compiles vital registration data from national authorities but may vary in completeness and accuracy across countries.^3^^,^^9^ For Denmark, Finland, Iceland, Norway, and Sweden, incidence data were sourced from NORDCAN, cancer statistics for the Nordic countries, which are derived from long-standing, high-quality national cancer registries.^10^ These datasets covered up to 2023, and we utilized the most recent 10-year data in this analysis. In total, incidence and mortality trend analyses included 46 and 63 countries, respectively. Trends were evaluated using estimated annual percent change (EAPC) over a ten-year interval. Further methodological details are provided in the Supplementary Methods, available via Mendeley at https://data.mendeley.com/datasets/yx7zzv7xdp/2.

### Statistical analysis (primary analysis)

We reported the absolute numbers of new cases and deaths, with crude rates (ie, age-standardized incidence rates and mortality rates per 100,000 people) calculated by the Segi World standard population. The cumulative risk of developing or dying of melanoma before 75 years of age was presented as a percentage, assuming no competing causes of death.^11^ The M:I ratio, defined as the number of deaths divided by the number of new cases, was calculated by world region as an indicator of melanoma fatality, recognizing its limitations as a proxy for survival in the absence of robust survival data.

### Forecasting the future burden

To assess the future melanoma burden, we applied age-specific incidence and mortality rates in 2022 to United Nations-projected populations for 2050. Scenarios were constructed as follows: (1) a baseline scenario assuming that the rates observed in 2022 remain constant through 2050, and (2) alternative scenarios assuming that these rates increase or decrease by 1% to 3% annually until 2050. Estimates were generated for 66 time points (2025 to 2050) and stratified by age group, country, region (United Nations Development Programme classification), and HDI level. Detailed modeling assumptions and analytic code are described in the Supplementary Methods, available via Mendeley at https://data.mendeley.com/datasets/yx7zzv7xdp/2.

### Estimating the UVR-attributable burden (secondary analysis)

Quantifying population-level exposure to UVR is challenging due to limited data on ambient levels and cumulative lifetime exposure. As no population is entirely unexposed to solar UVR, conventional methods using unexposed reference groups to estimate population attributable fractions (PAFs) are not applicable. Instead, we adopted an alternative approach used in previous studies,^7^^,^^12^^,^^13^ selecting sex-specific populations presumed to have minimal UVR exposure across all countries and years as a reference. Excess melanoma incidence in other populations was attributed to UVR, under the assumption that the reference populations represent a minimal effective exposure level and baseline risk. PAFs by sex were calculated for each country using the following formula:

$$PAF=\left(I_{p}-I_{u}\right)/I_{p}$$

where $I_{p}$ is the incidence in 2022 in the population of interest, and $I_{u}$ is the incidence in the reference population.

Sex-specific reference populations were selected using a quantile-based approach based on the distribution of age-standardized melanoma incidence rates across all countries and years in GLOBOCAN 2022. Specifically, we evaluated reference definitions corresponding to the lowest observed incidence and the first, third, and fifth percentiles. We adopted the fifth percentile incidence as the primary reference for PAF estimation to provide a conservative. The countries corresponding to each reference definition (lowest observed incidence and percentile-based benchmarks) are listed in Supplementary Table I, available via Mendeley at https://data.mendeley.com/datasets/yx7zzv7xdp/2. These populations were considered contextually plausible references due to phenotypic (dark skin pigmentation) and behavioral (culturally limited sun exposure^14^) protective factors (see Supplementary Methods and Table I for details, available via Mendeley at https://data.mendeley.com/datasets/yx7zzv7xdp/2). This approach was selected to maintain methodological consistency and comparability of PAF estimates across regions. PAF variances were estimated using the standard error of the reference incidence rates, with corresponding uncertainty intervals (UIs).

Furthermore, to forecast the future burden, we applied projected incidence rates from GLOBOCAN 2022 and used the same PAF approach to estimate UVR-attributable melanoma cases through 2050. All results were summarized globally, by region, and by HDI. All data analyses and visualizations were conducted using Python (version 3.10.4; Python Software Foundation).

### Role of funding source

The study funder had no role in the study design, data collection, data analysis, data interpretation, report writing, or decision to submit the manuscript for publication.

## Results

### Global burden of melanoma in 2022

In 2022, an estimated 330,000 (95% UI, 320,000-340,000) new melanoma cases and 59,000 (55,000-63,000) deaths occurred globally (Supplementary Table II, available via Mendeley at https://data.mendeley.com/datasets/yx7zzv7xdp/2), corresponding to 1.66% of all new cancer diagnoses and 0.60% of all cancer-related deaths. The global age-standardized incidence and mortality rates were 3.20 and 0.53 per 100,000 people, respectively.

**Fig 1:**
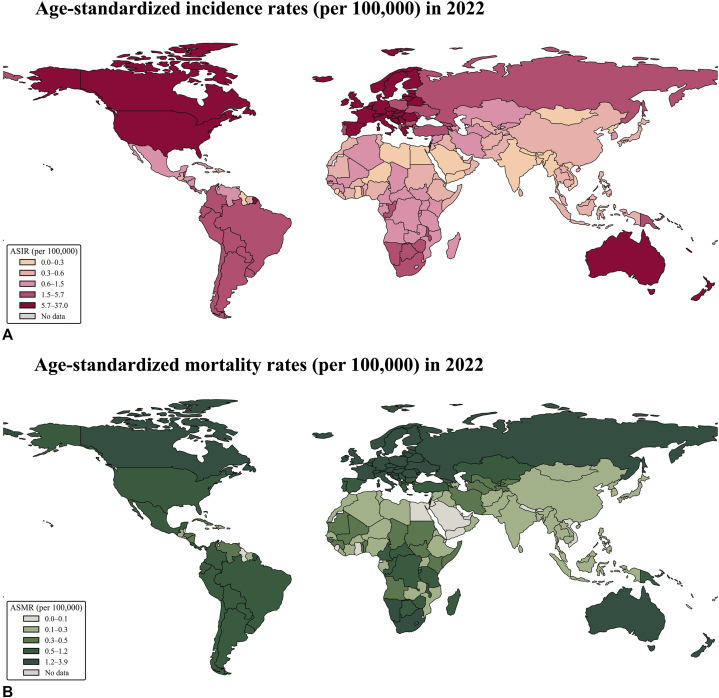
Age-standardized incidence and mortality rates (per 100,000 people) population of melanoma in 2022. **A,** Age-standardized incidence rates (per 100,000 people) of melanoma in 2022. **B,** Age-standardized mortality rates (per 100,000 people) of melanoma in 2022.

**Fig 2:**
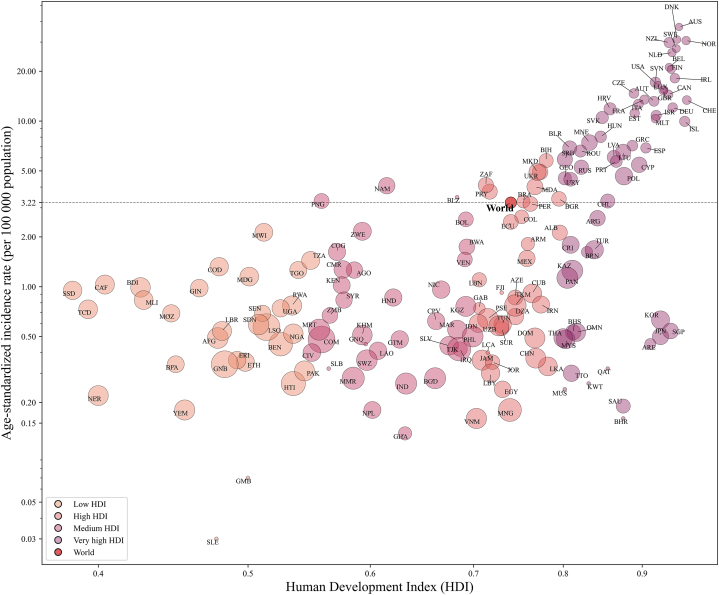
Regional disparities in age-standardized incidence and mortality rates (per 100,000 people) of melanoma by Human Development Index (HDI) and continent. ∗Each point represents a country, and the radius of each dot corresponds to the country’s mortality-to-incidence (M:I) ratio. ∗∗Corresponding ISO-3 codes are provided in Supplementary Table XIII, available via Mendeley at https://data.mendeley.com/datasets/yx7zzv7xdp/2.

Across regions, the age-standardized incidence rate ranged from 0.41 per 100,000 people in Asia to 29.80 per 100,000 people in Oceania, a 73-fold difference (Fig 1, *A*), whereas mortality varied more narrowly, from 0.21 to 2.30 per 100,000 people, respectively (Fig 1, *B*). The resulting M:I ratio was highest in Asia (51.22%) and lowest in Northern America (6.75%) and Oceania (7.72%). By HDI, M:I ratios generally declined with development, with the lowest values in very-high-HDI countries (11.58%), followed by high-HDI (36.67%), low-HDI (48.33%), and medium-HDI (51.35%) countries (Fig 2, Supplementary Fig 1, and Supplementary Table II, available via Mendeley at https://data.mendeley.com/datasets/yx7zzv7xdp/2).

**Fig 3:**
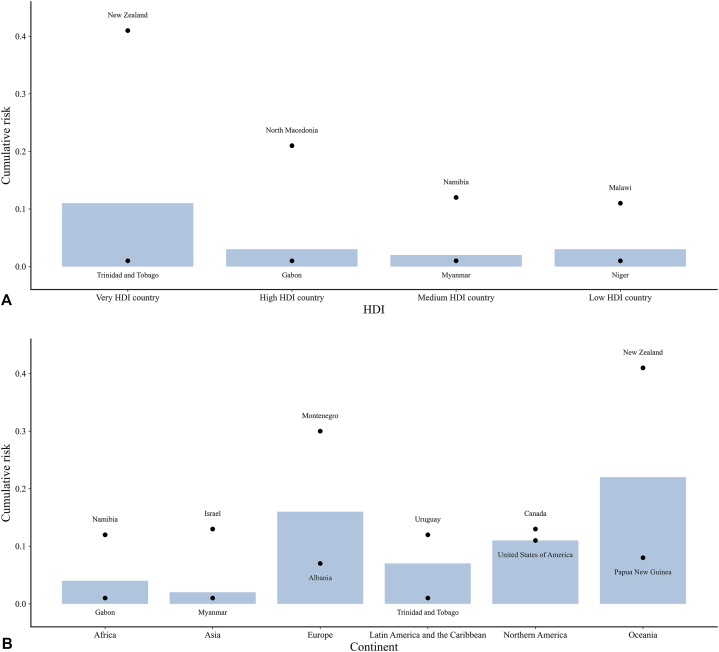
Cumulative risk of melanoma before the age of 75 years by **(A)** Human Development Index (HDI) and **(B)** continent.

Based on current rates, the lifetime risk of being diagnosed with melanoma was 0.35%, and the risk of death was 0.05% (Fig 3 and Supplementary Table III, available via Mendeley at https://data.mendeley.com/datasets/yx7zzv7xdp/2). Australia had the highest lifetime risk of melanoma diagnosis (4.02%) and one of the highest mortality risks (0.41%). Across HDI categories, lifetime risks of both diagnosis and death generally increased with higher HDI.

### Ten-year trends in incidence and mortality

**Fig 4:**
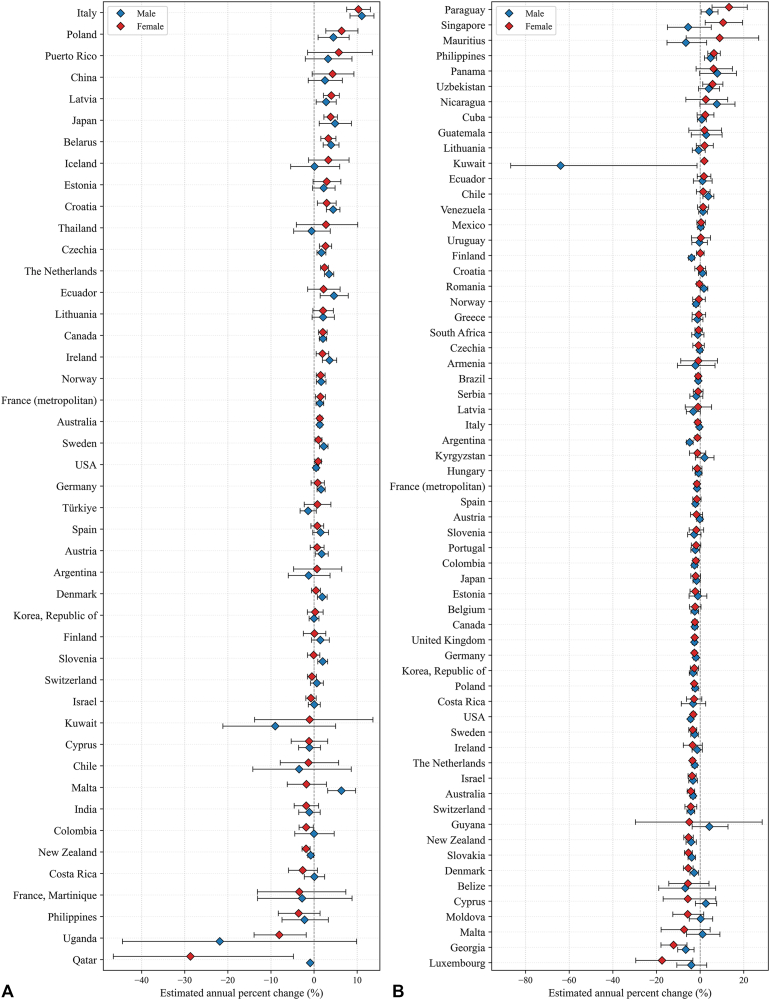
Most recent ten-year trends in melanoma **(A)** incidence and **(B)** mortality (95% UI).

Among 46 countries with ≥10 years of melanoma incidence data that met CI5plus quality standards, 19 (41.30%) exhibited significant increases in age-standardized incidence rates. Italy reported the highest EAPC for both sexes combined (10.62% [8.25-13.05]), followed by Poland (5.45% [2.51-8.48]) and Kuwait (4.79% [−8.22 to 19.64]; Supplementary Table IV, available via Mendeley at https://data.mendeley.com/datasets/yx7zzv7xdp/2). Countries with the highest HDI (≥0.85) largely exhibited increasing trends, whereas those with an HDI <0.85 tended to show declining trends, consistent across both sexes (Fig 4, *A*). By sex, incidence increased in 20 countries for males and in 16 countries for females. New Zealand was the only country with a significant decline in incidence among males (−0.78% [−1.44 to −0.12]), while declines among females were observed in Latvia (−28.69% [−46.60 to −4.79]), Uganda (−8.08% [−13.93 to −1.83]), New Zealand (−1.86% [−0.92 to −2.79]), and Colombia (−1.85% [−3.48 to −0.18]).

Among 63 countries with ≥10 years of mortality data, 4 (8.69%) showed increasing trends, most notably Paraguay (7.49% [3.06-12.10]), Philippines (5.34% [3.01-7.73]), and Uzbekistan (4.53% [0.59-8.63]; Supplementary Table V, available via Mendeley at https://data.mendeley.com/datasets/yx7zzv7xdp/2). Twenty seven countries (58.70%) had decreasing mortality rates, led by Georgia (−8.62% [−12.47 to −4.61]) and Luxembourg (−7.09% [−11.50 to −2.45]). Mortality generally declined or stabilized in countries with an HDI ≥0.85 and increased or remained stable in those with an HDI <0.85 (Fig 4, *B*).

### Burden attributable to UVR exposure

**Fig 5:**
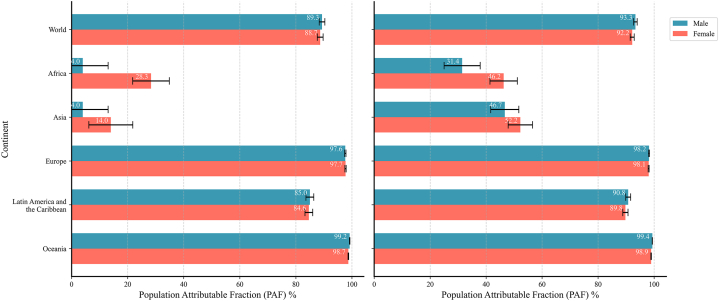
Burden attributable to ultraviolet radiation exposure by continent in 2022 and 2050 based on GLOBOCAN 2022 forecasts.

Fig 5 illustrates the proportion of melanoma cases attributable to UVR exposure globally and by region. Using the fifth percentile reference, UVR accounted for a greater proportion of melanoma cases among males (89.33% [88.33-90.34]) than females (88.68% [87.66-89.71]). Regionally, the burden of melanoma attributable to UVR was predominantly concentrated in Oceania, where nearly all cases were linked. In contrast, a very low burden of UVR-attributable burden was observed in Asia (males: 4.00% [0.00-13.02]; females: 28.33% [21.82-34.85]) and Africa (males: 4.00% [0.00-13.02]; females: 14.00 [6.18-21.82]; Supplementary Table VI, available via Mendeley at https://data.mendeley.com/datasets/yx7zzv7xdp/2).

### Projected burden and its attribution to UVR exposure

Assuming that age-specific rates remain constant at 2022 levels, an estimated 600,000 new melanoma cases and 117,000 deaths are expected in 2050, representing increases of 80.7% and 99.8% from 2022, respectively. By age, the burden is projected to decline sharply in those <30 years (−97.2% cases, −98.8% deaths) but remain stable or increase in those aged ≥70 years (−0.5% cases, +35.2% deaths). We presented estimated numbers of new cases and deaths up to 2050 by projection scenario in Supplementary Figs 2 and 3 and Tables VII-XII, available via Mendeley at https://data.mendeley.com/datasets/yx7zzv7xdp/2.

By 2050, the proportion of melanoma cases due to UVR will rise across all HDI levels, remaining highest in very-high-HDI countries (Fig 5). Males consistently have higher PAFs than females. The largest absolute PAF increases between 2022 and 2050 occur in low and medium-HDI countries. In medium-HDI countries, female PAFs rise from 65.0% to 76.7% (+11.7%) and male PAFs from 70.5% to 81.5% (+11.0%). In low-HDI countries, female PAFs increase by 7.0% and male PAFs by 5.2%.

## Discussion

### Key findings of this study

This study provides updated global, regional, and national estimates of melanoma burden and future projections attributable to UVR exposure, using the most recent GLOBOCAN 2022 data. First, using the fifth percentile reference, >88% of melanoma cases globally are attributable to UVR, disproportionately concentrated in Oceania. Second, projections to 2050 suggest striking increases in UVR-attributable melanoma in low- and medium-HDI regions, where preventive infrastructure remains limited. Third, melanoma incidence and mortality are declining in younger individuals but rising in those aged ≥70 years, reflecting aging demographics and cumulative UVR exposure. Fourth, M:I ratios are highest in Asian countries, reflecting inequities in early detection and care. Finally, our methodological refinement-using sex-specific, population-based UVR reference rates-enhances the precision and regional relevance of attributable burden estimates.

### Comparison with previous studies

Prior studies have consistently reported that a large proportion of melanoma cases are UVR-attributable, and that a substantial fraction may be preventable through reduced UVR exposure. A recent study using GLOBOCAN 2022 estimated the current global melanoma burden associated with UVR, particularly in Australia/New Zealand, Northern Europe, and North America, where lighter skin phenotypes are more prevalent.^8^^,^^12^ Two national-level studies provided insights for the United Kingdom^13^ and parts of Northern Europe^7^ but lacked global projections and stratification by age or HDI.

Our study extends prior work by using GLOBOCAN 2022 data from 185 countries to quantify both the current and future burden attributable to UVR. To our knowledge, this is the first analysis to project UVR-attributable melanoma incidence to 2050 by HDI groups. Importantly, while a previous study used an external birth cohort as a reference,^8^ we improved PAF estimation by using sex-specific reference populations with minimal UVR exposure, to improve internal validity and better account for biological and cultural variation in sun exposure patterns.^15^^,^^16^ Moreover, our projections draw attention to the emerging burden in low- and medium-HDI regions, historically underrepresented but expected to experience rapid increases.^11^^,^^17^

### Plausible mechanisms

UVR is a well-established cause of melanoma, inducing DNA damage and suppressing local immune responses. Intermittent, intense sun exposure-especially in early life-markedly elevates melanoma risk,^18^ particularly among fair-skinned individuals with less natural photoprotection.^19^ Historically, these factors have contributed to the high incidence in high-HDI countries, where recreational sun exposure is prevalent.^3^^,^^20^

While the current burden remains highest in high-HDI countries, projections suggest a shifting landscape. The growing burden in low- and medium-HDI countries likely reflects urbanization, westernized lifestyles, and more outdoor activity without adequate sun protection.^21^ In these regions, limited access to dermatologic care and public awareness exacerbate the burden.^22^ Notably, in parts of sub-Saharan Africa, melanoma mortality appears disproportionately high relative to reported incidence, consistent with a higher case fatality driven by delayed diagnosis and constrained access to timely diagnostics and treatment.^11^^,^^23^ Additionally, many low-HDI countries are located in high-UVR zones, and climate change and ozone depletion may further intensify exposure.^24^ Inadequate cancer surveillance may also obscure the true extent of the burden.^25^ Together, these factors suggest that melanoma prevention efforts must expand beyond high-income settings to avoid growing disparities. The projected sharp decline among individuals aged <30 years primarily reflects demographic shifts, as age-specific incidence rates were held constant in the projection model.

### Implications

With >88% of melanoma cases attributable to UVR, the vast majority are preventable. However, many low- and medium-HDI countries lack effective UVR protection policies and access to dermatologic care.^26^ Our projections indicate that these regions will face the largest relative increases by 2050, highlighting the need for targeted prevention strategies beyond high-income contexts.

This growing burden also challenges progress toward Sustainable Development Goal Target 3.4, which seeks to reduce premature mortality from non-communicable diseases (NCDs) by one-third by 2030. Cancer control plans should integrate UVR mitigation through school-based education, occupational regulation, and restrictions on indoor tanning.^22^^,^^27^^,^^28^ Clinically, our findings highlight the need for improved risk stratification, early detection, and diagnostic capacity in high-risk and resource-limited settings.^1^ Prevention remains the most effective strategy, and global coordination aligned with WHO’s NCD Action Plan is needed to mitigate the rising burden.

### Limitations

Our study has several limitations. First, the quality and completeness of GLOBOCAN 2022 data vary by country and HDI. In many low- and middle-HDI countries, cancer registries are incomplete or of variable quality, necessitating extrapolation and modeling, which introduces uncertainty, as reflected in wider UIs. Nevertheless, the application of harmonized methods and the inclusion of uncertainty intervals enhance cross-country comparability and provide the most precise and reliable estimates currently attainable at the global level. Second, melanoma may be underdiagnosed in populations with darker skin due to lower clinical suspicion and limited access, possibly underestimating the burden in parts of Africa, Asia, and small island states. Very low reported case numbers in these regions may reflect both true biological differences in susceptibility and disparities in cancer surveillance infrastructure. Accordingly, these patterns may not be fully generalizable to settings with different phenotypic distributions and health-system capacity. Third, subtype-specific estimates, such as those for acral lentiginous melanoma that is largely unrelated to UVR exposure, were not available in GLOBOCAN 2022, and thus could not be explicitly considered in our analysis. Accordingly, we retained the established approach for our primary analysis, which leverages a minimally exposed reference cohort without subtype-specific exclusions to preserve global comparability. This choice may overestimate the UVR-attributable fraction in settings where non-UVR-related subtypes are relatively more prevalent.^8^ Therefore, future work should incorporate subtype-specific adjustments where robust population-based data permit. Fourth, cumulative risk estimates were based on the net formulation, assuming no competing causes of death, which may underestimate risk in high-mortality settings. Fifth, the PAF estimation relied on reference populations presumed to have minimal UVR exposure, based on the lowest observed melanoma incidence across GLOBOCAN 2022. Low incidence may reflect phenotypic (eg, dark skin) or behavioral protection, and variation in screening intensity across countries could influence the reference baseline and corresponding PAF estimates. However, sensitivity analyses using alternative reference thresholds (first, third, and fifth percentiles) yielded similar results, supporting the robustness of our findings. Sixth, projections applied static or linearly adjusted rates to UN forecasts and may implicitly reflect past population-level trends in cumulative UVR exposure, but they do not explicitly model future behavioral, climatic, or demographic changes. Nonetheless, our aggregate-level approach is consistent with global burden estimation methods and remains essential for cross-national comparisons in the absence of harmonized individual-level exposure data. Finally, projections of the UVR-attributable burden were based on fixed 2022 PAFs, thereby assuming stable behaviors, climatic conditions, and public health interventions.

## Conclusion

In this population-based study with global modeling, >88% of melanoma cases were attributable to UVR, with the highest burden in very high-HDI countries and the fastest projected increases in lower-HDI settings. As the first study to generate stratified, long-term projections of UVR-attributable melanoma burden at the global level, this work offers a timely resource for policymakers and cancer control planners.

## Conflicts of interest

None disclosed.
